# Abdominal fat quantification using convolutional networks

**DOI:** 10.1007/s00330-023-09865-w

**Published:** 2023-07-12

**Authors:** Daniel Schneider, Tobias Eggebrecht, Anna Linder, Nicolas Linder, Alexander Schaudinn, Matthias Blüher, Timm Denecke, Harald Busse

**Affiliations:** 1 Department of Diagnostic and Interventional Radiology, Leipzig University Hospital, Liebigstr. 20, Haus 4, 04103 Leipzig, Germany; 2 Present Address: Innovation Center Computer-Assisted Surgery (ICCAS), University of Leipzig, Semmelweisstr. 14, 04103 Leipzig, Germany; 3 Integrated Research and Treatment Center (IFB) Adiposity Diseases, Leipzig University Medical Center, Philipp-Rosenthal-Str. 27, 04103 Leipzig, Germany; 4 Helmholtz Institute for Metabolic, Obesity and Vascular Research (HI-MAG) of the Helmholtz Center Munich at the University of Leipzig and University Hospital Leipzig, Philipp-Rosenthal-Str. 27, 04103 Leipzig, Germany

**Keywords:** Adipose tissue, Obesity, Image processing, computer-assisted, Magnetic resonance imaging, Deep learning

## Abstract

**Objectives:**

To present software for automated adipose tissue quantification of abdominal magnetic resonance imaging (MRI) data using fully convolutional networks (FCN) and to evaluate its overall performance—accuracy, reliability, processing effort, and time—in comparison with an interactive reference method.

**Materials and methods:**

Single-center data of patients with obesity were analyzed retrospectively with institutional review board approval. Ground truth for subcutaneous (SAT) and visceral adipose tissue (VAT) segmentation was provided by semiautomated region-of-interest (ROI) histogram thresholding of 331 full abdominal image series. Automated analyses were implemented using UNet-based FCN architectures and data augmentation techniques. Cross-validation was performed on hold-out data using standard similarity and error measures.

**Results:**

The FCN models reached Dice coefficients of up to 0.954 for SAT and 0.889 for VAT segmentation during cross-validation. Volumetric SAT (VAT) assessment resulted in a Pearson correlation coefficient of 0.999 (0.997), relative bias of 0.7% (0.8%), and standard deviation of 1.2% (3.1%). Intraclass correlation (coefficient of variation) within the same cohort was 0.999 (1.4%) for SAT and 0.996 (3.1%) for VAT.

**Conclusion:**

The presented methods for automated adipose-tissue quantification showed substantial improvements over common semiautomated approaches (no reader dependence, less effort) and thus provide a promising option for adipose tissue quantification.

**Clinical relevance statement:**

Deep learning techniques will likely enable image-based body composition analyses on a routine basis. The presented fully convolutional network models are well suited for full abdominopelvic adipose tissue quantification in patients with obesity.

**Key Points:**

*• This work compared the performance of different deep-learning approaches for adipose tissue quantification in patients with obesity.*

*• Supervised deep learning–based methods using fully convolutional networks  were suited best.*

*• Measures of accuracy were equal to or better than the operator-driven approach.*

**Supplementary Information:**

The online version contains supplementary material available at 10.1007/s00330-023-09865-w.

## Introduction

Body composition analysis aims to non-invasively categorize and quantify metabolically relevant tissues like fat, muscle, or bones. Over the years, radiological imaging data have become one of the obvious sources for such an analysis. More recently, convolutional neural networks have received widespread attention for automated image segmentation and tissue quantification due to the prospect of a substantially higher efficiency over manual and semiautomated methods [1].

Obesity is defined as the abundance of ectopic abdominal or subcutaneous adipose tissue. It is strongly associated with a variety of other diseases like diabetes, coronary heart disease, metabolic syndrome, and many types of cancer [2–4]. Body composition and especially obesity are commonly characterized by measuring body mass index or bioelectrical impedance. These measures, however, may not resolve individual fat depots, which is crucial for proper phenotyping [2, 5]. Likewise, dual-energy X-ray absorptiometry (DEXA) is only approximate, operator-dependent, and requires good patient compliance. With their high anatomical resolution in three dimensions, tomographic imaging techniques have become a de facto standard for the quantification of body fat. Computed tomography (CT) and magnetic resonance imaging (MRI) can therefore help to identify specific phenotypes of obesity.

In contrast to subcutaneous adipose tissue (SAT), visceral fat (VAT) has a distinct metabolic role [6, 7] and is strongly associated with a metabolic syndrome [8]. In comparison with CT, MRI provides the best soft tissue contrast but is less available and more demanding (time and effort). For prospective studies, MRI is preferred due to the lack of ionizing radiation and the overall lower risk profile. Fat amounts on MRI are usually identified by their high signal intensity in T1-weighted images and quantified by a number of approaches. Contrast agent is usually not required for body composition studies. Manual contouring [9] is often considered the reference standard but suffers from long processing times [10]. Semiautomated or supervised methods will save some time by automated computation of an approximate segmentation that is visually inspected and interactively adjusted in case of errors [10–12].

Fully automated methods are faster and eliminate any interreader variability [11–13] but may not necessarily provide the most accurate result [14]. They are typically based on geometrical modelling and image processing techniques like thresholding (e.g., fixed signal intensities for tissue discrimination), morphological operators, and clustering (e.g., the definition of background and foreground). An artificial neural network is trained with proper reference annotations from expert readers and might therefore meet the requirements of both speed and accuracy.

Over the past years, deep learning techniques have shown promising results promoting automation and personalization in a wide array of medical applications [15–19]. For complex medical image processing and analysis tasks including body composition assessment, convolutional neural networks have become a major method of choice [20, 21]. Tissue composition and distribution are analyzed via image segmentation techniques such as fully convolutional networks (FCNs) [22, 23]. Their hierarchical encoder-decoder structure identifies spatial associations in the input images and generates high-resolution segmentation masks. Accordingly, FCNs are also promising candidates for the quantification of abdominal fat tissue [24–26]. Variations of the UNet architecture have already proven to be highly suitable for medical image segmentation in general [15].

This work aimed to assess the performance of supervised deep learning-based methods for adipose tissue quantification from MR images—using three different FCNs based on the UNet architecture—and compare the results with a ground-truth semiautomated approach. It is characterized by the combination of a relatively large number of MRI datasets, full abdominopelvic coverage, and a deliberate selection of patients with obesity.

## Material and methods

### Patients and MRI

Whole abdominal MRI data were available from an IRB-approved single-center study at an academic research institution (Integrated Research and Treatment Center AdiposityDiseases, Leipzig University Medicine, Leipzig, Germany) investigating the long-term effects of strength versus endurance training on the cardiometabolic risk factors for patients with obesity (BMI ≥ 35 kg/m^2^) (trial registration number: NCT01435057). The dataset involved 331 MRI examinations and 12,422 abdominal MRI slices.

All patients had been examined in a 1.5-T MRI system (Achieva XR, Philips Healthcare) in a supine position using the integrated whole-body coil for signal reception. The main pulse sequence for fat quantification was a dual-echo gradient echo with these parameters: 50 transverse slices (two stacks covering the abdominopelvic region between the diaphragm and pubic symphysis), slice thickness 10 mm, interslice gap 0.5 mm, echo times 2.3 ms (opposed phase) and 4.6 ms (in phase), repetition time 76 ms, flip angle 70°, field of view 530 mm × 530 mm, acquisition matrix 216 × 177 and reconstruction matrix 480 × 480.

### Semiautomated segmentation

Reference segmentation was provided by two experienced readers who annotated all abdominal MRI slices with SAT and VAT amounts. A purely manual segmentation of all MRI slices (12,422) was not considered for this task because of the immense amount of time and effort. Instead, an in-house software framework, DicomFlex [16], was used for semiautomated segmentation of SAT and VAT amounts (area and volume) from these T1-weighted gradient-echo MR images using information from both in-phase and out-of-phase images. This method involved the computation and manual supervision of SAT and VAT regions of interest (ROI) as well as the supervised definition of a threshold for the histogram of MRI signal intensities separating fat and nonfat amounts within the VAT ROI [12]. Overall, this typically involved 30–40 individual slices and required 15 to 20 min for the whole dataset—roughly 30 s per slice.

This approach generated some regional misclassifications, for example, visceral fat inside the kidneys or intestines, because tissues showed a slight overlap for MRI signal intensities near the threshold. False visceral fat amounts are conspicuous in the images but their contribution to the overall VAT amount is typically very small. An experienced reader carefully inspected these regions and dynamically adjusted the threshold to visually balance between extra and omitted visceral fat amounts. In the following, the term fat quantification refers to methods involving image segmentation.

### FCN architectures and training

Three FCN architectures for fully automated SAT and VAT segmentation were implemented: UNet [17], DenseUNet [18, 19], and CDFNet [20, 21]. They all process input images with a chosen resolution of 256 × 256 pixels and generate segmentation maps of the same dimension. The dataset was composed of T1-weighted (in-phase) MR images as model input and corresponding ground truth segmentation maps as a target. All three models were evaluated using a five-fold cross-validation scheme with training, validation, and test subsets, respectively. Each FCN was trained in a supervised manner using the training subsets. The evaluation was carried out on the test subsets, while the validation sets were used to prevent overfitting. Using the described cross-validation scheme, FCN performance could be assessed on the entire dataset. Further technical details and flow charts of the architectures are provided in the supplementary information.

### Evaluation metrics

The pixelwise agreement between the adipose tissue segmentations of the FCN models and the ground truth was evaluated with the accuracy metric and the Dice similarity coefficient. The quantification of adipose tissue volumes was validated with a selection of aggregation metrics, each highlighting different aspects of the prediction performance. The Pearson correlation coefficient is a widely used measure of linear correlation and was used here to verify a strong correlation between true and predicted fat volumes. The mean percentage error is used to estimate the systematic error (bias) of the predictor. In addition, the standard deviation of the relative differences between true and predicted volumes provides the variational error of the predictions. Both error contributions may be combined to the root mean square percentage error. The similarity of the distributions between ground truth and predicted adipose tissue volumes may be estimated with the second Wasserstein distance. For better comparison with the error metrics, relative differences were also used here. Finally, the excess kurtosis was computed to assess the contribution of outliers (here, severely false predictions) to the aggregated metrics. The functional forms of the metrics used in this work may be found in the supplementary information.

## Results

**Fig. 1:**
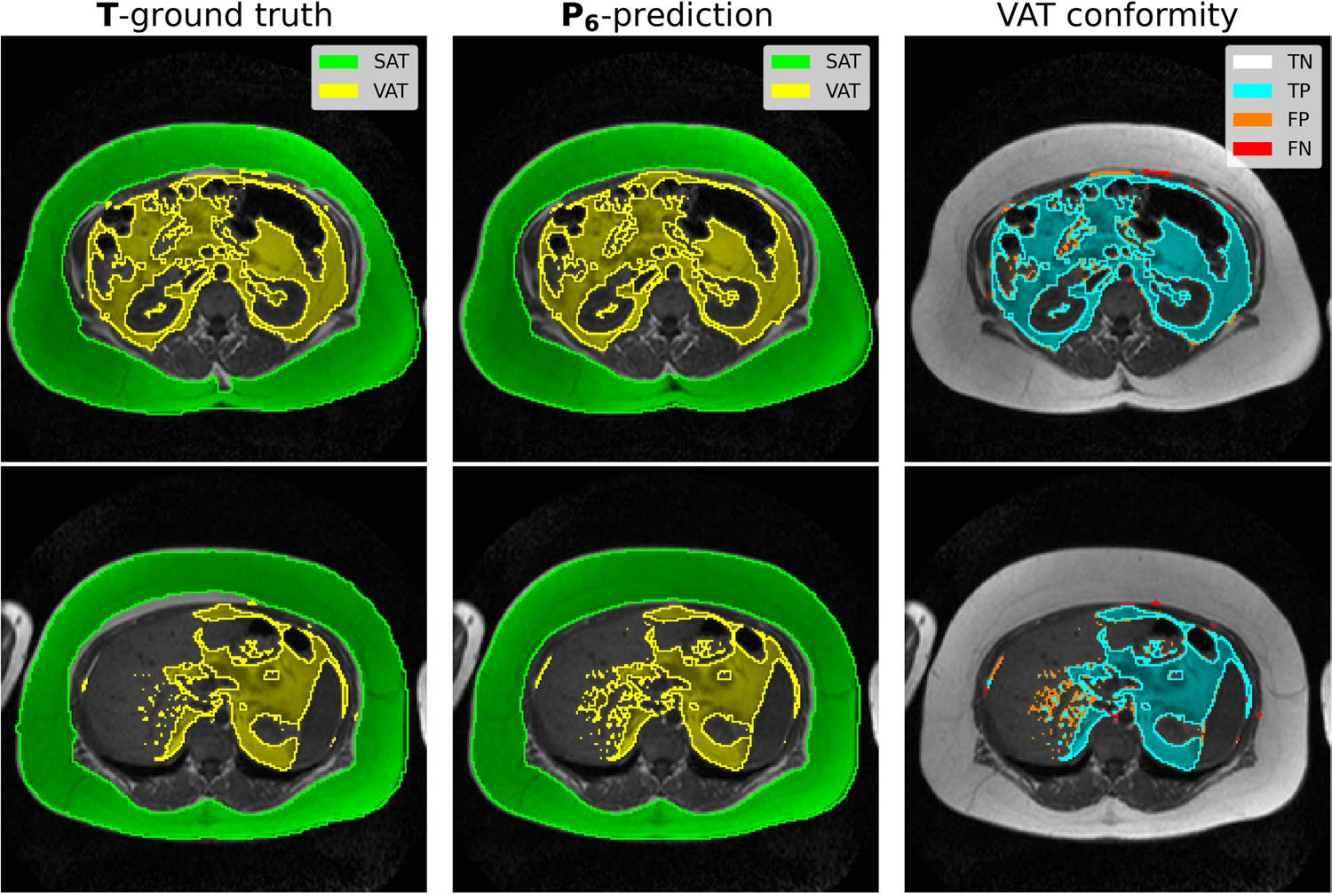
SAT and VAT segmentation for two sample MRI slices. The left column shows the ground truth $\mathbf{T}$, the center column the ${\mathbf{P}}_{6}$ prediction (obtained with the CDFNet and augmented training data), and the right column displays VAT agreement between the two annotations. TP: true positive; TN: true negative; FP: false positive; FN: false negative

Table 1 summarizes the agreement between predicted (${\mathbf{P}}_{\mathbf{i}}$) and ground-truth ($\mathbf{T}$) adipose tissue segmentation maps using the described cross-validation scheme. Each FCN architecture was trained with and without data augmentation. SAT classifications were generally more accurate than VAT predictions. The agreement with the ground truth was marginally higher when augmented data was added during training. This effect was more pronounced for VAT. Pixelwise similarity measures for DenseUNet and CDFNet are slightly higher than their counterparts corresponding to the vanilla UNet architecture. Figure 1 shows two sample MR images after CDFNet (${\mathbf{P}}_{6})$ segmentation in comparison with the ground truth.

**Table 1:** Average pixel-wise segmentation performance of the FCN models in cross-validation

Label	Model		Accuracy	Dice score	
	Architecture	Augmentation		SAT	VAT
*P*_1_/*T*	UNet	N	0.975	0.949	0.870
*P*_2_/*T*	UNet	Y	0.977	0.953	0.883
*P*_3_/*T*	DenseUNet	N	0.975	0.949	0.876
***P***_**4**_**/*T***	DenseUNet	Y	**0.978**	**0.954**	**0.889**
*P*_5_/*T*	CDFNet	N	0.976	0.949	0.879
***P***_**6**_**/*T***	CDFNet	Y	**0.978**	**0.954**	**0.889**

Comparison of segmentation maps (first column) between adipose tissue annotations ${{\varvec{P}}}_{{\varvec{i}}}$ from different FCN architectures (second column) and ground truth ${\varvec{T}}$ – without or with data augmentation (third column) during training. Average accuracy (ACC) and Dice scores (DSC) for SAT and VAT. Boldface values highlight the overall best scores

**Fig. 2:**
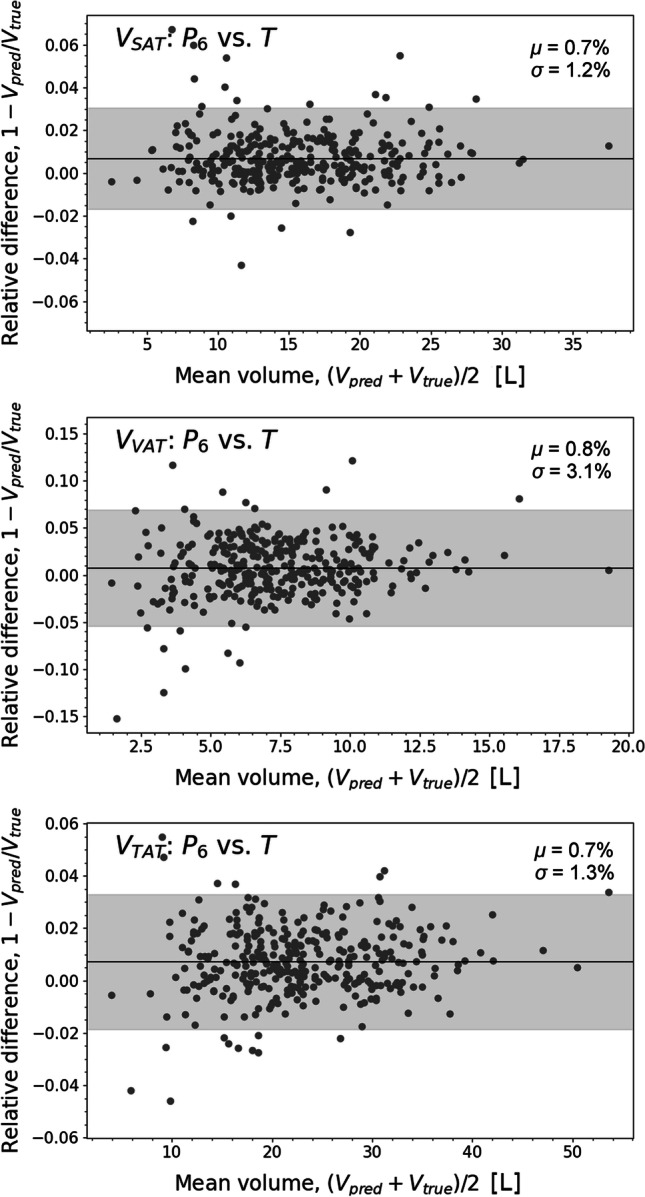
Bland–Altman plots for SAT (top), VAT (middle), and TAT (bottom) volumes between **P**_6_ and **T.** The fat volumes ${\mathrm{V}}_{\mathrm{pred}}$ in $\mathrm{L}$ (liters) were obtained from the predicted SAT and VAT segmentations **P**_6_**,** while the volumes ${\mathrm{V}}_{\mathrm{true}}$ were derived from the ground truth annotations **T***.* Bland–Altman plots were annotated with mean relative difference $\upmu$ (black line) and 95% confidence range (light gray area)

Table 2 provides an overview of the performance of the FCN models in adipose tissue quantification. Strong agreement was found between the predicted and ground truth SAT volumes, as revealed by a Pearson correlation of 0.999 and a 1–2% RMSPE largely comprised of variation error. In comparison, FCN model predictions of VAT volumes showed correlation coefficients of up to 0.997 and the lowest RMSPE of 3.2%. Figure 2 shows the Bland–Altman plots of ${\mathbf{P}}_{6}$ adipose tissue volume predictions. Bland–Altman plots for total adipose tissue volume (TAT) and VAT/SAT volume ratio can be found in the supplementary material.

**Table 2:** Patient-wise fat quantification performance metrics of the FCN models ${{\varvec{P}}}_{{\varvec{i}}}$ in cross-validation

Compartment	Label	*R*	*MPE*	*SD*	*RMSPE*	*PW*_2_	*k*_ex_
**SAT**	*P*_1_/*T*	**0.999**	0.017	0.020	0.026	0.020	13.7
	*P*_2_/*T*	**0.999**	0.010	0.014	0.017	0.012	4.1
	*P*_3_/*T*	**0.999**	0.007	0.019	0.020	0.013	15.4
	*P*_4_/*T*	**0.999**	**0.006**	**0.012**	**0.014**	**0.009**	3.9
	*P*_5_/*T*	**0.999**	0.009	0.015	0.018	0.012	3.6
	***P***_**6**_**/*T***	**0.999**	0.007	**0.012**	**0.014**	**0.009**	4.4
**VAT**	*P*_1_/*T*	0.996	0.012	0.049	0.051	0.028	7.0
	*P*_2_/*T*	0.996	0.017	0.032	0.037	0.021	2.3
	*P*_3_/*T*	0.996	0.017	0.053	0.055	0.031	28.9
	*P*_4_/*T*	0.996	0.014	0.033	0.035	0.020	2.1
	*P*_5_/*T*	0.995	0.020	0.036	0.041	0.027	5.5
	***P***_**6**_**/*T***	**0.997**	**0.008**	**0.031**	**0.032**	**0.016**	3.4

See Table 1 for model details. Pearson correlation coefficient $R$; mean percentage error $MPE,$ standard deviation of the relative sample errors $SD$; root-mean-squared percentage error *RMSPE*; second Wasserstein distance of relative sample errors ${PW}_{2}$ and excess kurtosis of relative differences *k*_*ex*_. Boldface values highlight the overall best scores

Table 3 summarizes the variability for the best FCN predictions (${\mathbf{P}}_{6}$) and ground truth for both SAT and VAT (cross-validation with 331 patients) as well as that between two readers (SAT, selection of 29 patients). For reference purposes only, corresponding values from an independent previous assessment are also given. The average fat segmentation times for one MRI slice are reported in Table 4. With the FCN models, whole-abdominal adipose tissue quantification, assuming a few tens of MRI slices per patient, are on the order of a few seconds on a PC with a powerful graphic card. On a standard “office” PC, processing may take 1–2 min. For comparison, semiautomatic segmentation required 15 to 20 min for the whole dataset–roughly 30 s per slice.

**Table 3:** Variability of various methods for fat quantification

Segmentation method	SAT		VAT	
	ICC	CV	ICC	CV
*P*_6_/*T* [present study]	**0.999**	**0.014**	0.996^*^	0.031^*^
*T*_2_/*T*_1_ [present study]	0.996	0.024	–	–
manual [8]	0.995	0.018	0.972	0.069
supervised automated [8]	0.997	0.021	0.997^*^	0.029^*^

Agreement between adipose tissue volumes predicted by the best FCN model (**P**_**6**_) and ground truth **T** during cross-validation (331 patients) is shown in the first row. The second row presents the consensus between two different operators (SAT only, selection of 29 patients). The bottom rows report variability between two readers for SAT and VAT quantification from an independent, previous assessment using a similar MRI protocol and semiautomated Matlab tool [8]. *ICC*, intra-class correlation; *CV*, coefficient of variation

Boldface values highlight the overall best scores (* indicates similar best values)

**Table 4:** Average compute times of one forward pass (one MRI slice) of the FCN during inference (segmentation of one image with 256 × 256 resolution) on CPU (Intel Xeon E5-2637 v2) or GPU (Nvidia GeForce GTX TITAN Black). For comparison, segmentation of a single slice with the semiautomated method (used for ground truth annotations) required roughly 30 s per slice

Model	CPU time	GPU time
	[sec/slice]	[sec/slice]
UNet	0.74	0.05
DenseUNet	1.34	0.09
CDFNet	1.05	0.09

## Discussion

Adipose tissue volume is a common biomarker for various clinical outcomes. This work evaluated to what extent fully convolutional networks may serve to automatically segment and quantify abdominal adipose tissue from MR images. Whole-abdominal image data of 331 study patients were annotated for SAT and VAT amounts using a semiautomated segmentation method. Three deep learning architectures, UNet and its derivatives DenseUNet and CDFNet, were trained on the original patient data with additional samples generated by randomized image transformations. All models were evaluated for segmentation and fat quantification performance in cross-validation.

Although variation error remains the biggest source of deviation, all models tend to slightly underestimate VAT volume likely due to class imbalance (i.e., commonly smaller VAT volumes as compared to SAT and remaining tissue). For both types of adipose tissue, the low Wasserstein distance indicates the preservation of the ground-truth volume distribution. The moderate excess kurtosis suggests a relatively consistent quantification performance among the samples.

Overall, data augmentation with tuned hyperparameters improved model performance. DenseUNet and CDFNet turned out to be more efficient than the original UNet implementation with similar or better results (despite using fewer parameters) at the cost of increased processing time. The highest evaluation measures for adipose tissue volume analysis were achieved by the CDFNet trained on the original and augmented data (${\mathbf{P}}_{6}$).

The FCN-based methods showed excellent SAT segmentation and quantification accuracy, and the corresponding agreement for VAT was very good. A comparison with the inter-rater agreement from user-guided quantification showed the superiority of the FCN models for SAT, and similar to higher performance for VAT as opposed to manual annotation alone or supervised image processing.

Agreement between methods was generally higher for SAT than for VAT, which is attributed to the high variability of VAT with respect to distribution, size, and shape. VAT amounts need to be carefully distinguished from surrounding structures and organs, such as the bowel, pancreas, or urinary bladder [22–24]. VAT quantification is already challenging for human segmentation as indicated by the lower inter-rater reliability [12].

The intraclass correlation and coefficient of variation of the FCN approach are also in line with the variability reported between two expert readers using an earlier Matlab tool (similar semiautomated approach) as well as a widely used commercial software (manual segmentation) [12]. A more detailed assessment is not possible here, because the above parameters were obtained by different readers on different subjects.

With FCN processing times between a few seconds and about a minute (depending on the hardware), whole-abdominal fat amounts (tens of slices) may be segmented practically on the fly [25]. As of now, our FCN-based approach is at least one order of magnitude faster than the semiautomated method used for ground truth **T** segmentation here (15–20 min per patient for 30–40 slices). These results demonstrate the promising reliability of FCN approaches in the quantification of adipose tissue compartments.

One of the first reports on the automated segmentation of visceral and subcutaneous adipose tissue featured a very small sample size (around 40) and showed good agreement with Dice coefficients between 0.82 and 0.92 [26]. In a later work, a higher level of agreement (> 0.95) was reached with an improved neural network [27]. That study was limited by a small amount of training data and lacked external validation. Proper external validation was performed in a more recent work but the sample size remained low with only 20 cases [28].

In general, the lack of sufficiently large datasets is still regarded as a major limitation of current approaches [29]. An open-source design might assist with the distribution and acceptance of the methods [30]. Modern implementations have reached a new level of processing speed with times on the order of 1 min [21]. Whole-body adipose tissue analyses are also offered as commercial services, but the associated fees might limit their use to smaller batches [31].

This work has a number of limitations. As already mentioned in the methods section, the ground truth for adipose tissue volumes was not established by manual segmentation due to time constraints. We accepted the disadvantage of local misclassifications (see Fig. 1, bottom row), which were “learned” by the FCN, and decided to take advantage of a much higher number of patients with reference data.

On a technical level, our supervised learning methods focus on point estimation of adipose tissue volumes only. The presented FCN has no means of reporting its reliability on out-of-distribution data and should always be professionally supervised by an expert reader.

Various methods have been proposed for uncertainty estimation for deep learning in general and body composition analysis in particular [28, 32], which should be considered for future research. Additionally, ground truth annotations should ideally be made by several independent observers to estimate the uncertainty of label generation as well.

The trained model should be able to extrapolate to similar cohorts and imaging systems without any retraining. Even within a given cohort, data outliers may arise from different acquisition parameters, imaging artifacts, or rare phenotypes. One of the key challenges of deep-learning approaches is the availability of proper training data. Here, methods using generative adversarial learning schemata [33, 34] or spatially aware optimization strategies may be employed to improve generalization to unseen data. Future work needs to analyze the performance of the trained fat quantification models on external cohorts.

In conclusion, this work demonstrates that deep-learning approaches for adipose tissue quantification from MRI data are also feasible for patients with obesity. The resulting accuracy was equal to or better than that of operator-driven approaches with processing requiring substantially less time and effort.

### Supplementary Information

Below is the link to the electronic supplementary material.

Supplementary file1 (PDF 733 KB)

## Abbreviations

CDFNet: Competitive dense fully convolutional network
FCN: Fully convolutional network
ROI: Region of interest
SAT: Subcutaneous adipose tissue
VAT: Visceral adipose tissue

## Declarations

### Guarantor

The scientific guarantor of this publication is Harald Busse, PhD.

### Statistics and biometry

No complex statistical methods were necessary for this paper.

### Study subjects or cohorts overlap

MRI data of the cohort have been previously used for intraindividual analyses only evaluating different approaches to the estimation of adipose tissue amounts. All these studies did not consider nor analyze any diagnostic information of the subjects related to the original clinical investigation (registration number: NCT01435057) on the long-term effects of strength versus endurance training on cardiometabolic risk factors for patients with obesity (BMI ≥ 35 kg/m^2^).

### Methodology

- retrospective
- observational
- performed at one institution
