# Supplementary material for: Abdominal fat quantification using convolutional networks
Source: Eur Radiol. 2023 Jul 12;33(12):8957–64. doi: 10.1007/s00330-023-09865-w (PMC10667157; doi:10.1007/s00330-023-09865-w)

SUPPLEMENTARY INFORMATION

Evaluation Metrics

With $i \in \mathbb{N}_{\leq n}$ indexing a set of *n* samples, consider a quantity Y with ground-truth realizations $\{y_i\}$ and a predictor $\hat{Y}$ with predictions $\{\hat{y}_i\}$. In classification (discrete Y), the predictor may be assessed with the *accuracy* metric

$$ACC(Y, \hat{Y}) = \frac{1}{n} \sum_{i=1}^{n} \delta_{y_i \hat{y}_i}$$

or the class-wise *Dice similarity coefficient*

$$DSC(Y, \hat{Y}, c) = \frac{2 \sum_{i=1}^{n} \delta_{c y_i \hat{y}_i}}{\sum_{i=1}^{n} (\delta_{c y_i} + \delta_{c \hat{y}_i})},$$

using Kronecker delta δ_{kl} and a category identifier c. For the segmentation task, $y_i, \hat{y}_i, c \in \{\mathrm{SAT}, \mathrm{VAT}, \mathrm{other}\}$. Here, accuracy and dice similarity coefficient are used to measure the pixelwise agreement between predicted and ground-truth adipose tissue.

The following metrics may be used for predictors of continuous random variables Y, e.g., quantization of adipose tissue volumes. The *Pearson correlation coefficient*

$$R(Y, \hat{Y}) = \frac{\sigma_{Y\hat{Y}}}{\sigma_Y \sigma_{\hat{Y}}}$$

measures the linear correlation between true and predicted values assuming standard deviations σ_Y and $\sigma_{\hat{Y}}$, and covariance $\sigma_{Y\hat{Y}}$.

Consider the relative difference $D = 1 - \hat{Y}/Y$ with realizations $\{d_i\}$. The *mean percentage error* $MPE(D) = \mu_D$ is a measure of the systematic prediction error (bias) with μ_D being the arithmetic mean of relative difference D. The *standard deviation* of the relative differences $SD(D) = \sigma_D$ estimates the variation prediction error by eliminating

the mean bias from the individual differences. The *root-mean-square percentage error* is a measure of average total prediction error according to *RMSPE*$(D) = \mu_{D^2}^{\frac{1}{2}}$, where $RMSPE^2 = MPE^2 + SD^2$.

The *second Wasserstein distance* PW_2 measures the similarity between the predicted and ground truth distributions. Here, the *relative* difference was used as cost function to facilitate the comparison with *RMSPE*. PW_2 is obtained by

$$PW_2(Y, \hat{Y}) = \left\langle \sum_{j=1}^{n} f_{ij} \left(1 - \frac{\hat{y}_i}{y_j}\right)^2 \right\rangle^{\frac{1}{2}},$$

with chevron brackets ⟨⟩ indicating the arithmetic mean, and the set $\{f_{ij}\}$ representing the solution of the following linear assignment problem,

$$\begin{aligned} &\min_F(PW_2) \\ &f_{ij} \in \{0,1\} \\ &\sum\nolimits_{i=1}^{n} f_{ij} = 1\ \forall\ j \\ &\sum\nolimits_{j=1}^{n} f_{ij} = 1\ \forall\ i, \end{aligned}$$

which can be solved, for instance, with the Hungarian algorithm. PW_2 is bounded by $0 \leq PW_2 \leq RMSPE$ with 0 indicating similar distributions of predicted and true values.

Finally, the *excess kurtosis k* quantifies the excess contribution of the tails in the distribution of variation errors in comparison to the normal distribution and indicates the relevance of outliers. It is obtained via

$$k_{\text{ex}} = \frac{\mu_D^{(4)}}{\sigma_D^4} - k_{\mathcal{N}},$$

where $\mu_D^{(4)} = \mu_{(D-\mu_D)^4}$ denotes the fourth central moment and $k_{\mathcal{N}} = 3$ is the kurtosis of the standard normal distribution.

In this work, Pearson correlation coefficient, mean percentage error, standard deviation of the relative differences, root-mean-square percentage error, second Wasserstein distance, and excess kurtosis were used to validate the predictors of SAT and VAT volumes for a given patient.

FCN architectures

The UNet architecture (Fig. S1) is composed of an encoding pathway hierarchically capturing spatial context over different length scales and a symmetric decoder for proper resolution output (1). The encoder contains a series of convolution and downsampling (here: max pooling) operations. The decoding path is comprised of repeated convolution and upsampling sequences. Transposed convolutions are used for trainable interpolation during upsampling. Additional paths for gradient flow are provided by skip connections. The last convolution operation maps the resulting feature maps to the class logits. The UNet used here had a total of 8.8 million trainable parameters.

The DenseUNet architecture (2,3) (Fig. S2) is a slight variation of the UNet design. The number of encoder and decoder blocks were increased by one. All encoding and decoding sequences use three interconnected convolutional layers (4). A deterministic max-unpooling operation was chosen to compensate for the increased block complexity during upsampling. The number of feature channels was decreased to 64 and kept constant throughout the network. This lowered the numbered of learned parameters to 3.3 million.

Model training

Data were composed of T1-weighted (in-phase) MR images and their corresponding ground-truth segmentation maps. All FCN were trained in a supervised learning scheme using stochastic gradient descent with learning rate $l_r = 0.001$, momentum $m = 0.9$, and batch size $N_b = 4$ using cross-entropy loss as objective function. A common five-fold cross-validation scheme was applied to evaluate segmentation and fat quantification performance. For each split, the dataset was divided into training, validation and test subsets according to the ratio 3:1:1. In this way the entire dataset may be used for model evaluation, testing each of the five obtained models on the respective hold-out data and aggregating performance measures over all models. The training subset was shuffled prior to each epoch and early stopping was used for regularization (5), using, after each training epoch, model performance on the validation subset as indicator. At last, the hold-out test subset was used for model validation.

Several FCN were trained using data augmentation via random image transformations to improve generalization performance (6). This included affine transformations (rescaling, translation, rotation and shear), piecewise affine transformations, changes in perspective, pixel-intensity scaling as well as Gaussian blur, crop-and-pad and cutout (7). The corresponding hyperparameters of the transformations for data augmentation together with SGD parameters and CE-loss class weights were tuned in ex-ante cross-validation.

Lastly, the addition of competitive learning (8) turns the above DenseUNet into a CDFNet (Competitive Dense Fully Convolutional Network, Fig. S3). Instead of fully connecting the convolutions within encoding or decoding blocks, only the strongest

activations in each encoder and decoder sequence were allowed to pass (maxout operation). The same rule is applied to the skip connections between encoding and decoding pathways. It is hypothesized that a higher selectivity in features will improve the accuracy of the model (9). The CDFNet has 64 feature channels and 2.5 million trainable parameters.

Supplementary Figures

Figure S1. UNet architecture. Feature maps are shown in green with dimensional information: number of channels × number of pixels in first image dimension × number of pixels in second image dimension. Operations are shown as rounded rectangles. For convolutional layers, the kernel size is shown with $x \times y$ and the input and output channels with $C:D$. For each convolution, the padding was adjusted so that the input and output dimensions remain constant.

Figure S2. DenseUNet architecture. See Fig. S2 legend for details.

Figure S3. CDFNet architecture. See Fig. S2 legend for details.

References

1. Ronneberger O, Fischer P, Brox T (2015) U-Net: Convolutional Networks for Biomedical Image Segmentation. Available via http://arxiv.org/pdf/1505.04597v1. Accessed on 21 Apr 2023
2. Roy AG, Conjeti S, Navab N, Wachinger C (2018) QuickNAT: A Fully Convolutional Network for Quick and Accurate Segmentation of Neuroanatomy. Available via http://arxiv.org/pdf/1801.04161v2. Accessed on 21 Apr 2023
3. Cai S, Tian Y, Lui H, Zeng H, Wu Y, Chen G (2020) Dense-UNet: a novel multiphoton in vivo cellular image segmentation model based on a convolutional neural network. Quant Imaging Med Surg 10:1275–1285.
4. Huang G, Liu Z, van der Maaten L, Weinberger KQ (2016) Densely Connected Convolutional Networks. Available via http://arxiv.org/pdf/1608.06993v5. Accessed on 21 Apr 2023
5. Yao Y, Rosasco L, Caponnetto A (2007) On Early Stopping in Gradient Descent Learning. Constr Approx 26:289–315.
6. Shorten C, Khoshgoftaar TM (2019) A survey on Image Data Augmentation for Deep Learning. J Big Data 6.
7. DeVries T, Taylor GW (2017) Improved Regularization of Convolutional Neural Networks with Cutout. Available via http://arxiv.org/pdf/1708.04552v2. Accessed on 21 Apr 2023
8. Estrada S, Lu R, Conjeti S, et al (2020) FatSegNet A Fully Automated Deep Learning Pipeline for Adipose Tissue Segmentation on Abdominal Dixon MRI. Magn Reson Med 83:1471–1483.
9. Estrada S, Conjeti S, Ahmad M, Navab N, Reuter M (2018) Competition vs. Concatenation in Skip Connections of Fully Convolutional Networks. In: Shi Y, Suk H-I, Liu M (eds). Machine Learning in Medical Imaging. Cham: Springer International Publishing. pp. 214–222.

Figure S1. UNet architecture. Feature maps are shown in green with dimensional information: number of channels × number of pixels in first image dimension × number of pixels in second image dimension. Operations are shown as rounded rectangles. For convolutional layers, the kernel size is shown with $x \times y$ and the input and output channels with $C{:}D$. For each convolution, the padding was adjusted so that the input and output dimensions remain constant.

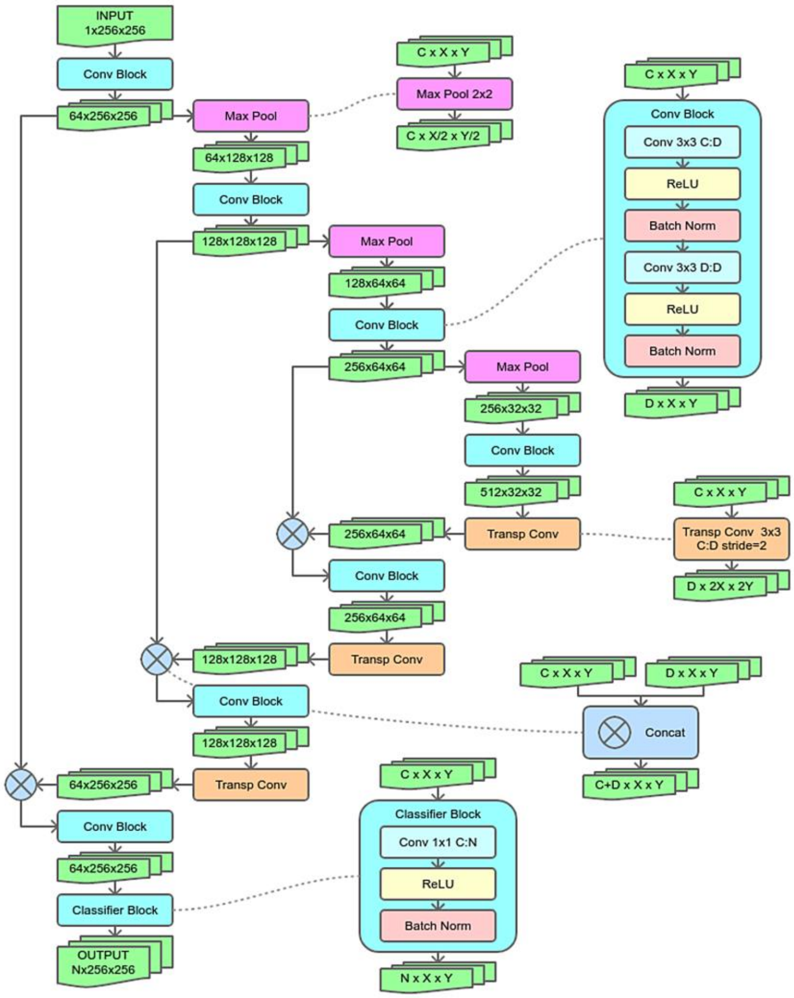

Figure S2. **DenseUNet architecture.** See Fig. S2 legend for details.

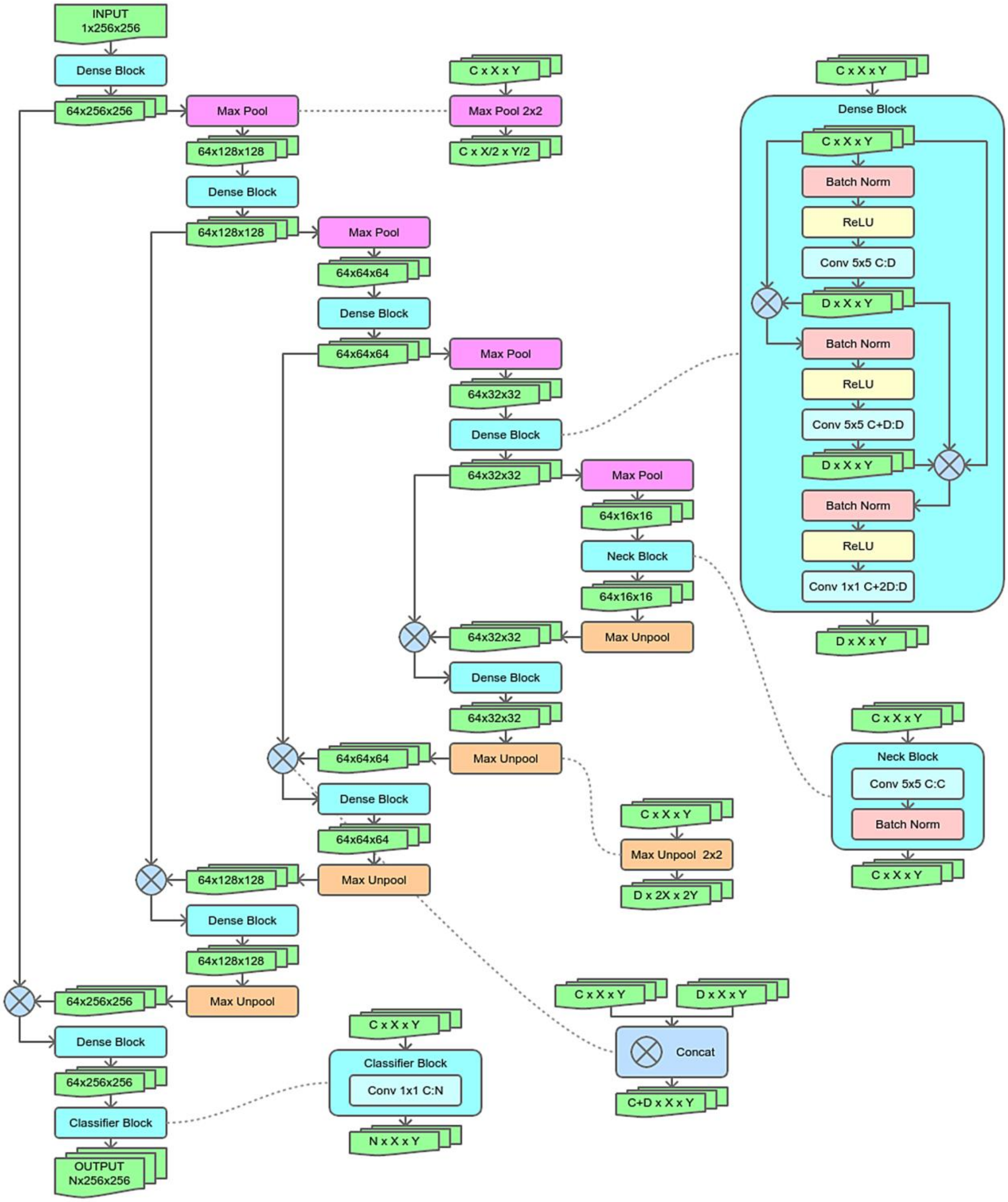

Figure S3. CDFNet architecture. See Fig. S2 legend for details.

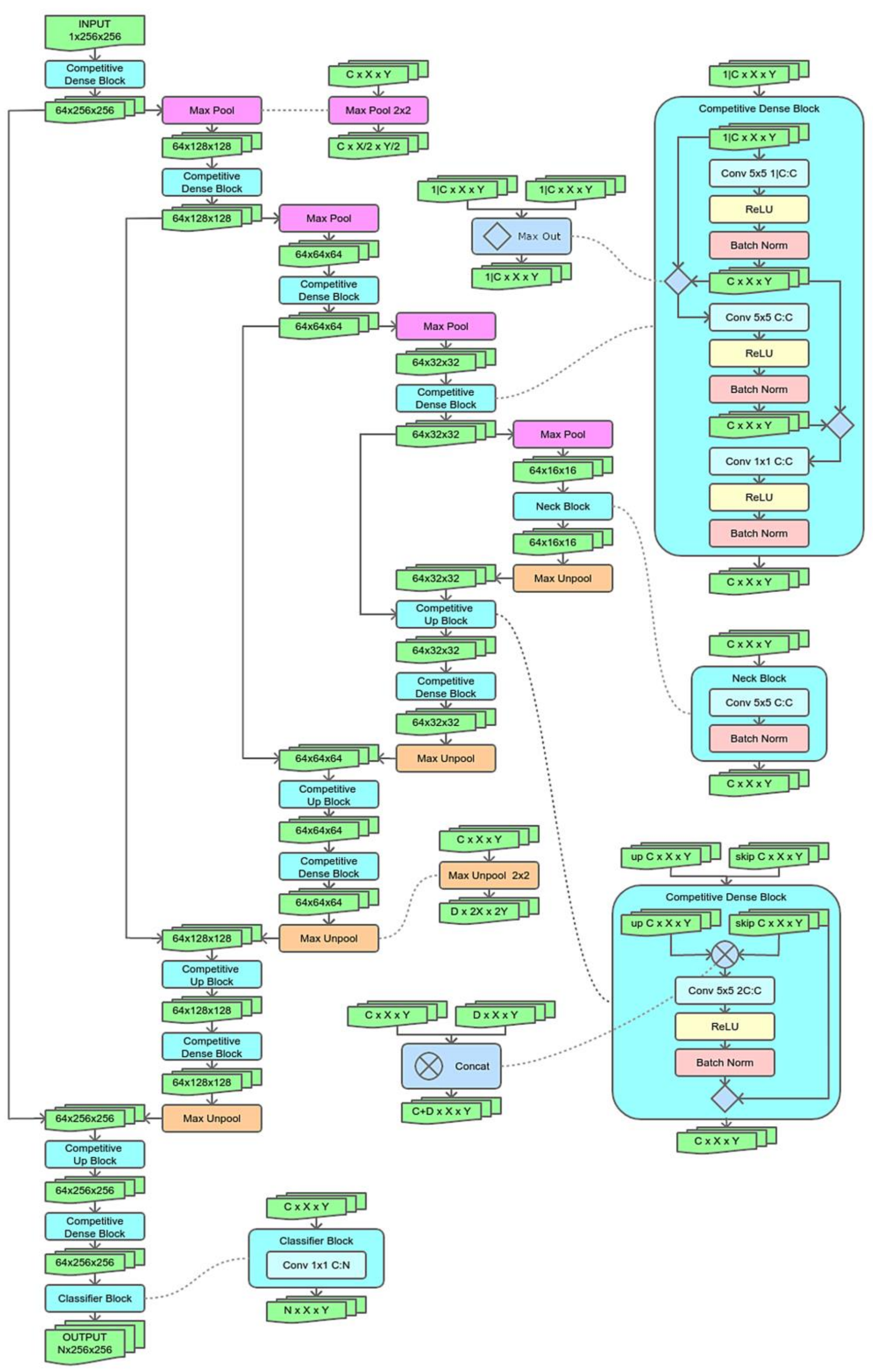

Supplement: Supplementary file 1 — Supplementary file1 (PDF 733 KB) [file 330_2023_9865_MOESM1_ESM.pdf]
